# The Effects of Vagus Nerve Stimulation on Animal Models of Stroke-Induced Injury: A Systematic Review

**DOI:** 10.3390/biology12040555

**Published:** 2023-04-05

**Authors:** Mohammad Yusuf Hasan, Rosfaiizah Siran, Mohd Kaisan Mahadi

**Affiliations:** 1 Centre for Drug Herbal and Development, Faculty of Pharmacy, Universiti Kebangsaan Malaysia, Jalan Raja Muda Abdul Aziz, Kuala Lumpur 50300, Malaysia; 2 Neuroscience Research Group (NRG), Faculty of Medicine, Jalan Hospital, Universiti Teknologi MARA, Sungai Buloh Campus, Sungai Buloh 47000, Malaysia

**Keywords:** vagus nerve stimulation, angiogenesis, apoptosis, forelimb strength, infarct volume, inflammation, neurological deficit score

## Abstract

**Simple Summary:**

Ischemic stroke is one of the most common causes of death worldwide, putting a significant strain on society and the healthcare system. In recent years, significant progress has been made in the treatment of ischemic stroke. However, owing to the ever-increasing cost of medical care, there is an urgent need to develop alternative treatments for stroke prevention and treatment. Vagus nerve stimulation, currently approved for the treatment of depression and epilepsy, has emerged as a useful therapeutic approach in recent decades. Although numerous animal studies have been conducted on vagus nerve stimulation, particularly in stroke conditions, little effort has been made to examine them together. In this review, we compiled and compared the findings of VNS studies up to June 2022. Vagus nerve stimulation was found to improve most of the stroke parameters examined. We concluded that, specifically in an animal model, vagus nerve stimulation may be a treatment option that helps alleviate stroke symptoms. These findings may be beneficial for future translational research in animal models and patients with stroke.

**Abstract:**

Ischemic stroke is one of the leading causes of death worldwide, and poses a great burden to society and the healthcare system. There have been many recent advances in the treatment of ischemic stroke, which usually results from the interruption of blood flow to a particular part of the brain. Current treatments for ischemic stroke mainly focus on revascularization or reperfusion of cerebral blood flow to the infarcted tissue. Nevertheless, reperfusion injury may exacerbate ischemic injury in patients with stroke. In recent decades, vagus nerve stimulation (VNS) has emerged as an optimistic therapeutic intervention. Accumulating evidence has demonstrated that VNS is a promising treatment for ischemic stroke in various rat models through improved neural function, cognition, and neuronal deficit scores. We thoroughly examined previous evidence from stroke-induced animal studies using VNS as an intervention until June 2022. We concluded that VNS yields stroke treatment potential by improving neurological deficit score, infarct volume, forelimb strength, inflammation, apoptosis, and angiogenesis. This review also discusses potential molecular mechanisms underlying VNS-mediated neuroprotection. This review could help researchers conduct additional translational research on patients with stroke.

## 1. Introduction

Ischemic stroke refers to a cerebral hypoperfusion caused by a thrombus blocking an artery or an embolic event in which debris from any other part of the body blocks the blood flow and is associated with local nutrient/oxygen deficiency [1,2]. The thrombolic event is usually secondary to atherosclerotic disease, arterial dissection, fibromuscular dysplasia, or an inflammatory condition, while the embolic event is caused by a cardiac event, such as atrial fibrillation or valvular disease [2]. This condition accounts for more than 85% of all stroke types, with varying symptoms depending on the location and severity of the brain tissue injury [1]. Symptoms include long-term disability that interferes with the patient’s daily activities, such as loss of limb function, loss of self-care abilities, and speech and language difficulties.

During a stroke, brain cells begin to die unless nearby blood vessels can deliver sufficient oxygen-rich blood to the ischemic area. The gold standard for ischemic stroke management is to restore the blood flow to the affected brain area as soon as possible after the attack, ideally within 4.5 h. Management can be accomplished using thrombolytic medications or a mechanical thrombectomy to remove the clot source causing the brain blockage [3,4]. However, because of the very narrow therapeutic window, over 69% of patients with stroke cannot receive immediate revascularization treatment, making neurological recovery difficult [5,6]. Combining revascularization therapy with other interventions, such as a non-pharmacological approach, may improve neurological outcomes in patients suffering from stroke [7].

The vagus nerve, also known as the pneumogastric nerve, is a parasympathetic branch of the autonomic nervous system that primarily regulates the rest and digest responses. Electrical stimulation applied directly to the vagus nerve trunk or dermatome with the vagal innervation nerve provides therapeutic benefits in various conditions. The use of vagus nerve stimulation (VNS) as an adjunct treatment for refractive epilepsy, recurrent depression, migraine, and cluster headache has been approved by the United States Food and Drug Administration (USFDA) [8,9]. Furthermore, mounting preclinical evidence suggests that VNS alone has a strong neuroprotective effect in animals with induced stroke [10]. The mechanism by which VNS improves stroke symptoms is not well understood. However, in general, several pathways have been proposed to be activated by VNS. The brainstem integration centre nucleus tractus solitarius (NTS) is activated when electrical stimuli are applied to areas with vagal nerve innervation. The vagal-NTS neural pathway stimulates the hypothalamus-pituitary-adrenal axis, causing anti-inflammatory cortisol to be released. The NTS also feeds signals to the vagus dorsal motor nucleus and the nucleus accumbens for vago-vagal and vago-sympathetic reflexes, resulting in neuro-immune interactions to inhibit inflammation [11]. In relation to stroke, the vagus nerve potentially regulates the cholinergic anti-inflammatory pathway via seven nicotinic acetylcholine receptors (α7nAChR) [12]. Stimulating the α7nAChR improves the inflammation regulated by microglial activation, thus protecting neuronal cells in the brain from oxidative stress [12,13].

VNS has been shown to be beneficial in stroke-induced animal models over the years [14,15]. Systematic reviews on the neuroprotective effects of VNS in randomised human trials have been published, with VNS being suggested to be effective and safe for post-stroke recovery [14,16,17]. However, there has been little effort to systematically analyse and compare the common physiological outcomes across animal studies. This is important because experimental conditions in animal studies are easier to control, eliminating any confounding variables that could influence the results and allowing for more conclusive and accurate interpretation. [18]. This systematic review aimed to summarize the neurological and behavioral findings from studies conducted on stroke-induced animal models with VNS as an intervention. The factors examined included infarct volume, neurological deficit score, forelimb strength, and inflammatory cytokine level. This could shed light on how VNS elicits neuroprotective effects in patients after a stroke, providing a resource for researchers to conduct additional translational research on patients with stroke.

## 2. Materials and Methods

### 2.1. Literature Search or Data Strategies

A systematic literature search was conducted to identify articles that reported a relationship between VNS and cerebral ischemic stroke in animal models. The Preferred Reporting Items for Systematic Reviews and Meta-analysis statement (PRISMA) and Cochrane Handbook for Systematic Reviews of Interventions were used to guide this systematic review [19]. Articles published until June 2022 were searched in Google Scholar, PubMed, Ovid, Scopus, Springer Link, and the Web of Science databases. The search was performed using the following keywords (vagus nerve stimulation OR transcutaneous vagus nerve stimulation OR vagus nerve) AND (ischemic stroke OR focal cerebral ischemia OR stroke) AND (animal models OR rat model OR mice model). Owing to the complexities of the research methods in delivering electrical stimuli, transcutaneous or direct stimulation was considered for VNS.

Some studies were hand-searched from the reference list of the retrieved articles.

We imported all articles from the search results into the reference manager Endnote, and any duplications were removed.

### 2.2. Inclusion and Exclusion Criteria

We included all studies published in English that reported the relationship between VNS and cerebral ischemic stroke injury, recovery, and rehabilitation in animal models. Articles that met the following criteria were considered unsuitable for this study:

- The focus was not ischemic stroke
- They reported on therapeutic interventions other than VNS and rehabilitation
- They consisted of a review article, an abstract, a conference paper, or a book chapter
- They were published in a language other than English
- They were missing main parts of the text

### 2.3. Data Extraction and Analysis

Duplicates were removed first, and full-text articles were independently reviewed by two reviewers (M.Y.H. and M.K.M.) who screened titles and abstracts based on inclusion and exclusion criteria to eliminate invalid records. When there was disagreement about an article, a third reviewer (R.S.) was consulted. Two authors (M.Y.H. and M.K.M.) extracted the data, including study author names, year of publication, study characteristics, potential neuroprotective outcome measures, and potential mechanisms of action.

The effects of VNS on ischemic stroke in animal models were classified into five categories: neurological deficit score, infarct volume, forelimb strength, inflammatory markers, angiogenesis, and apoptosis. The datasets obtained from the screening were heterogeneous, including both the experimental design and reported outcome. Therefore, it would be inappropriate to pool the data through a meta-analysis. Mean difference and 95% confidence interval were used to analyze the results, and the comparisons were visualized using forest plots. The primary outcomes and predictors of prognosis for ischemic stroke were reported as the neurological score, infarct volume, and forelimb strength in the majority of included studies. For comparison purposes, differences in rating scales between studies (that is, neurological deficit score: 4-point, 5-point, 12-point, 18-point, and 20-point scales) were converted to a 4-point scale. Articles with insufficient data were excluded from further analysis. Descriptive statistics were used to examine secondary outcomes, such as inflammatory markers, angiogenesis, and apoptosis.

### 2.4. Quality Assessment

The Office of Health Assessment and Translation (OHAT) Risk of Bias Rating Tool for Animal Studies [20] was used to check the data credibility or publication bias of the included studies. Articles were classified as having either a low risk of bias, a possibly low risk of bias, or a possibly high risk of bias (Table 1). One author (M.Y.H.) independently evaluated the bias, which was validated by another author (M.K.M.). Methodological reports of primary neuroprotective outcomes in each study were used to assess study quality.

### 2.5. Risk-of-Bias Assessment

The OHAT evaluation method was used to assess the possibility of bias owing to the study design. The OHAT method aids in identifying the bias risk in individual studies and internal validity in animal studies. [20]. We assessed all articles in the study for selection bias, detection bias, attrition or exclusion bias, and selective reporting bias using the OHAT criteria.

The following questions were asked in our study in order to assess the selection bias:

1. Was the induction of stroke ischemia and VNS treatment adequately randomized?
2. Was the allocation to study groups adequately concealed?

To assess performance bias, the following questions were asked:

3. Were the research personnel and animal subjects blinded to the study group?

To assess detection bias, the following questions were asked:

4. Can we be confident in the exposure characterization?
5. Can we be confident in the outcome assessment?

To assess attrition or exclusion bias, the following question was asked:

6. Was outcome data complete without attrition or exclusion from the analysis?

To assess selective reporting bias, the following question was asked:

7. Were all measured outcomes reported?

The risk of bias was defined using the following criteria: “definitely low” risk of bias to “definitely high” risk of bias [20].

- For rating the “definitely low” risk of bias, there should be ample evidence of “low risk” of bias practices from the study. For example, if the risk of bias due to selection bias was rated as “definitely low,” there should be direct evidence that animals were assigned to any study group using adequate and proper randomization descriptions.
- “Probably low” risk of bias is rated when there is an indirect evidence of low risk of bias practices. This is rated based on the magnitude and direction of deviation from a high risk of bias. For example, if a selection bias was rated as “probably low” risk of bias, it means that the study’s authors stated that allocation was random but did not describe the method used for randomization.
- “Probably high” risk of bias is rated for a study when there is insufficient information or information not provided about relevant risk-of-bias practices. In the case of selection bias, for example, there is insufficient information provided about how animals were assigned to the study groups.
- “Definitely high” risk of bias can be rated when there is direct evidence of high risk-of-bias practices. For example, if a selection bias is rated as having a definitely high risk of bias, it means that there is direct evidence that animals were allocated to study groups using a non-random method such as the investigator’s judgement, the results of a laboratory test, or there was a lack of a concurrent control group, indicating that randomization did not cover all study groups.

## 3. Results

**Figure 1:**
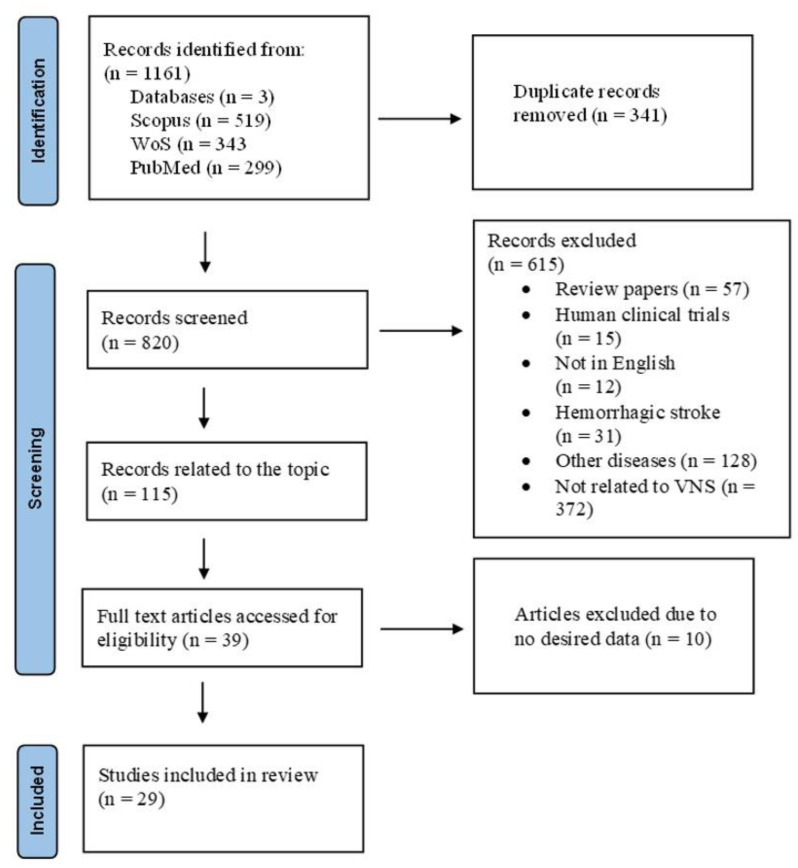
The systematic review process. The flow diagram outlines the search and selection process that was applied in the systematic review of published studies. All review steps were based on the PRISMA guidelines. Records (n) relate to the number of database searches, the number of abstracts screened, and the number of full texts obtained.

In this systematic review, we provided insights into the use of VNS as a therapeutic tool for the treatment of ischemic stroke. Duplicates were removed from the 1161 identified records, and records that were not published in English, were incomplete, or constituted a review were screened out. A total of 39 full-text articles were evaluated, with 29 relevant articles selected for review (Figure 1).

### 3.1. Bias Analysis

There was a low risk of selection bias in 23 of 29 studies. A high risk of performance bias was detected in two of the studies. All studies had a moderate risk of attrition/selection bias, except for one. We detected a moderate risk of detection bias in 14 studies, a high risk of detection bias in six studies, and the remaining studies had a definitely low risk of bias. Furthermore, six studies showed a moderate risk of selective reporting bias, and 23 showed a low risk of reporting bias (Table 1).

### 3.2. Characteristics of Included Studies

The characteristics of the 29 included studies are summarized in Table 2. All studies examined rodents, including one mouse and 28 rats of both sexes. Eight studies used a permanent middle cerebral artery occlusion model (PMCAO), four studies used a transient middle cerebral artery occlusion model (TMCAO), two studies utilised a filament occlusion model, seven studies used middle cerebral artery occlusion along with reperfusion (MCAO/R), while six studies used endothelial-1 injection. Zhao et al. (2022) [29] utilized a Longa thread embolization stroke model and Jiang et al. (2014) [30] used the intraluminal occlusion technique to induce stroke. The VNS intensity was consistent in most animal studies, with a frequency of 20–25 Hz, 5 mA, and 30 s per VNS stimulation every 5 min continuously for 1 h. Five studies stimulated the vagus nerve at a frequency of 30 Hz and a 0.8 mA current. Sixteen studies stimulated the right vagus nerve and 13 stimulated the left vagus nerve. Ten studies used VNS noninvasively, whereas 19 used it invasively.

**Figure 2:**
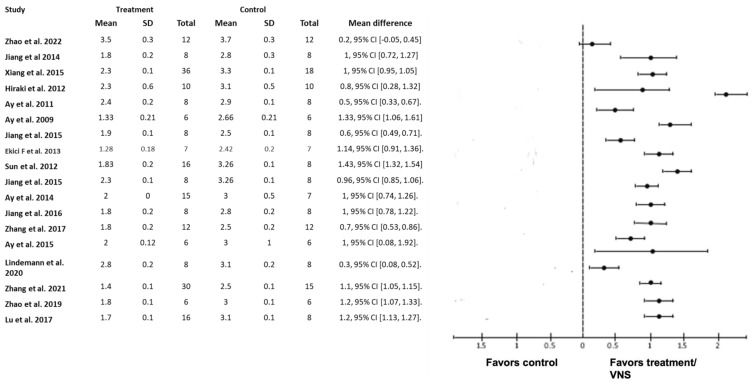
Forest plot: the outcome of vagus nerve stimulation (VNS) on neurological deficit score. The effect of each study (“favors control”, “favors treatment”, or “no effect”) was estimated through the standardized mean difference (horizontal axis). The dashed vertical line at 0 indicates that the study was determined to have “no effect”. The horizontal bars in each chart plot represent the mean difference with a 95% confidence interval for each study [21,22,23,24,25,26,27,28,29,30,31,32,35,38,39,40,41,42,49].

**Figure 3:**
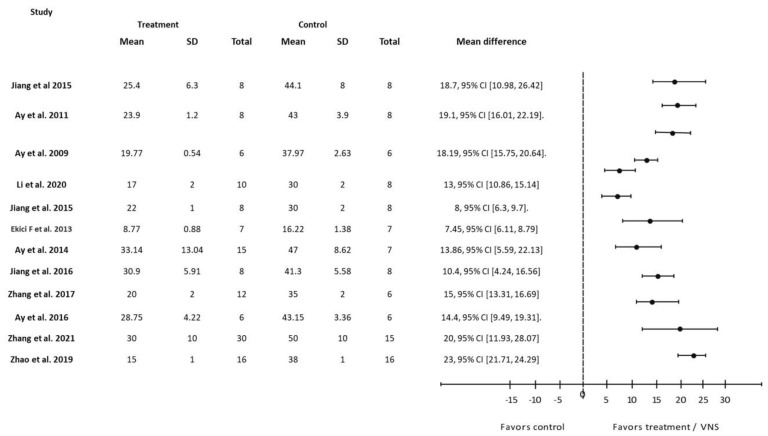
Forest plot: the outcome of vagus nerve stimulation (VNS) on infarct size reported as percentage reduction (%). The effect of each study (“favors control”, “favors treatment”, or “no effect”) was estimated through the standardized mean difference (horizontal axis). The dashed vertical line at 0 indicates that the study was determined to have “no effect”. The horizontal bars in each chart plot represent the mean difference with a 95% confidence interval for each study [22,23,26,32,34,35,37,38,39,41,42,49].

**Figure 4:**
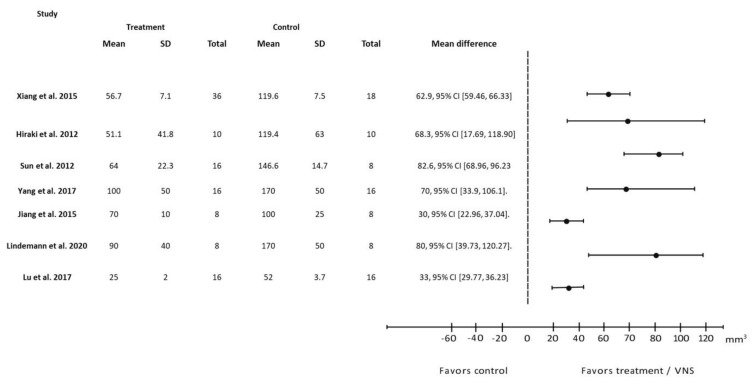
Forest plot: the outcome of vagus nerve stimulation (VNS) on infarct volume reported in mm^3^. The effect of each study (“favors control”, “favors treatment”, or “no effect”) was estimated through the standardized mean difference (horizontal axis). The dashed vertical line at 0 indicates that the study was determined to have “no effect”. The horizontal bars in each chart plot represent the mean difference with a 95% confidence interval for each study [21,24,25,26,31,34,40].

**Figure 5:**
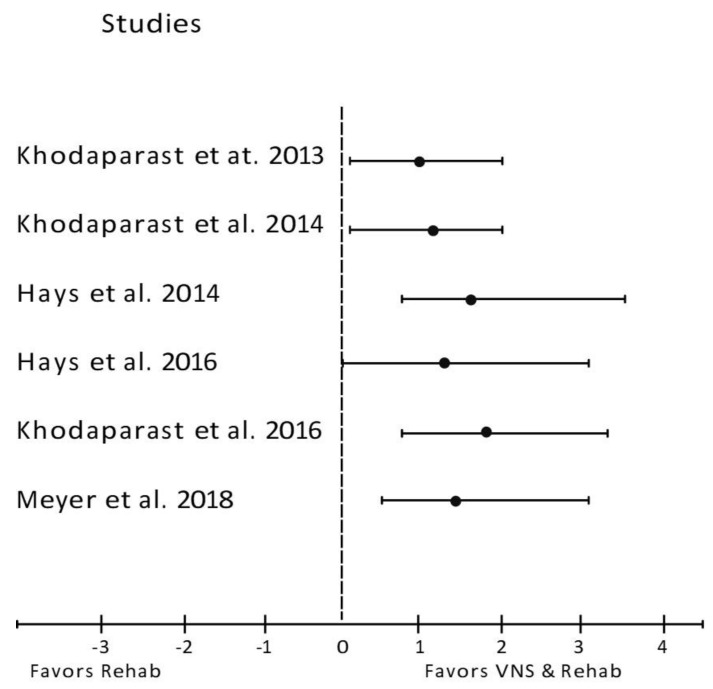
Forest plot: the outcome of vagus nerve stimulation (VNS) on forelimb strength. The effect of each study (“favors Rehab”, “favors VNS & Rehab”, or “no effect”) was estimated through the standardized mean difference (horizontal axis). The dashed vertical line at 0 indicates that the study was determined to have “no effect”. The horizontal bars in each chart plot represent the mean difference with a 95% confidence interval for each study [43,44,45,46,47,48].

In general, study outcomes were classified as prognostic predictors such as neurological deficit score (Figure 2), infarct size (Figure 3), infarct volume (Figure 4) and forelimb strength (Figure 5), in addition to the effects of VNS on inflammatory markers, angiogenesis, and apoptosis (Table 3).

**Table 2:** Characteristics of the included studies.

Author and Year	Sex and Species	Sample Size	Parameters Assessed	Stroke Model	Stimulation Parameter	Positioning of Electrode	Fundamental Finding
Xiang et al., 2015 [21]	Male Wistar rats	36	Neurological deficit score, infarct volume, and pro-inflammatory cytokines	PMCAO	20 Hz, 0.5 mA, 0.5 ms pulse width	Right cervical vagus nerve.	Applying 60 min VNS protects against cerebral ischemia by an anti-inflammatory mechanism neuroprotective effect is associated with the inhibition of expression of TNF-α and IL-6
Ay et al., 2015 [22]	Male Wistar rats	14	Neurological deficit score, cerebral infarct volume	PMCAO	0.5 mA, 0.5 ms pulse width, 20 Hz	Left cavum concha	Electric stimulation of the vagus nerve dermatome in the external ear activates brainstem afferent vagal nuclei and reduces infarct volume in rats.
Ay et al., 2016 [23]	Male spontaneously hypertensive rats	8	Neurological deficit score, infarct volume, microglial markers	PMCAO	25 Hz, 1 ms, 5 kHz, 12 V sine waves 2-min trains.	Overlying skin over the right vagus nerve.	Transcutaneous VNS reduces tissue injury and functional deficits while activating the vagal relay center in the brain. This was linked to a reduction in brain inflammatory responses (i.e.,: Iba-1, CD68. TNF-α).
Yang et al., 2018 [24]	Spontaneous hypertensive rats	12	Blood brain barrier integrity, neuronal cell death, infarct size.	PMCAO	25 Hz, 1 ms, 5 kHz, 15 V sine waves	Overlying skin over the right vagus nerve.	The neuroprotective role of transcutaneous VNS administrations during cerebral occlusion was investigated, and it was discovered that it spatially correlates with blood brain barrier integrity protection and infarct extent reduction.
Lindemann et al., 2020 [25]	Male Wistar rats	8	Cerebral blood flow, infarct volume, neurological deficit score, sensorimotor function	PMCAO	25 Hz, 0.5 mA, 0.3 ms pulse width	Overlying skin over the left vagus nerve.	Both VNS interventions limit the spread of cortical depolarization, resulting in a smaller stroke volume and improved motor outcome.
Zhang et al., 2021 [26]	Sprague-Dawley rats	16	TLR4/NFkB pathway, inflammatory cytokines	PMCAO	0.5 mA, 20 Hz, 0.5 ms pulse width	Left cervical vagus nerve	TLR4/MyD88/NF-kB-dependent polarization of microglia towards M2 is mediated by VNS.
Lu et al., 2017 [27]	Male Sprague-Dawley (SD) rats weighing	8	neurological deficits score, infarct volume, a7nAChR expression biomarkers	PMCAO	0.5 mA, 0.5 ms pulse width, 20 Hz	Left cervical vagus nerve	The application of VNS resulted in neuroprotection against ischemic injury, as well as anti-inflammatory responses. Inhibiting a7nAchR expression levels resulted in a significant worsening of neurological dysfunction, as well as an increase in cerebral infarct volume.
Long et al., 2022 [28]	Male Sprague–Dawley rats	15	White matter remyelination, ischemic volume, angiogenesis and the inflammation responses	PMCAO	2 mA, 0.5 ms pulse width, 20 Hz	Left cavum concha	VNS treatment improves dysphagia by promoting angiogenesis, remylination, and inhibiting inflammatory responses in white matter.
Zhao et al., 2022 [29]	Male Sprague Dawley rats	12	Neurological deficit score, inflammatory markers	Longa thread embolization	10 Hz, 1 mA, 0.5 ms pulse width	Left cymba concha	Seven days of auricular VNS promotes locomotor function recovery and inhibits IL-1, IL-6, and TNFα in the ischemic penumbra
Jiang et al., 2014 [30]	Male Sprague Dawley rats	8	Neurological deficit score, infarct volume, neuronal apoptosis, pro-inflammatory cytokines	Intraluminal occlusion technique	20 Hz, 0.5 mA, 0.5 ms pulse width	Right cervical vagus nerve.	60 min of VNS provides neuroprotection against acute cerebral I/R injury by suppressing inflammation and apoptosis in the ischemic penumbra. This was linked to cholinergic and α7nAchR/Akt pathway activation
Hiraki et al., 2012 [31]	Male Sprague-Dawley rats	10	Neurological deficit score, infarct volume	TMCAO	20 Hz, 0.5 mA, 0.5 ms pulse width	Right cervical vagus nerve.	The experimental evaluations were compared after 24 h, 48 h, 1 day, 2 days, and 3 days. VNS improves neurological functional scores and the severity of an ischemic lesion. This effect lasts for three weeks.
Ay et al., 2011 [32]	Male Wistar rats	8	Cerebralblood flow, infarct volume, neurological deficit score	TMCAO	0.5 mA, 30s, 0.5 ms, 20 Hz	Right and left cervical vagus nerve.	Both stimulation sides produced acute ischemic injury protection that was not mediated by changes in cerebral blood flow.
Zhang et al., 2017 [49]	Male Sprague–Dawley rats	12	Neuronal apoptosis, Neurological deficit score, infarct volume	TMCAO	0.5 mA, 0.5 ms pulse width, 20 Hz	Right cervical va-gus nerve	L-PGDS levels increased in rats treated with VNS in the peri-infarct region. L-PGDS may play a role in VNS’s suppression of the apoptotic response to ischemic damage.
Jiang et al., 2015 [34]	Male Sprague–Dawley rats	8	Infarct volume, neurologic deficit score, neuronal apoptosis	TMCAO	0.5 mA, 0.5 ms pulse width, 20 Hz	Right cervical vagus nerve	MiR-210 expression is increased in stimulated animals and has been linked to neuroprotection. The beneficial effects of VNS were reduced with miR-210 knockdown.
Ay et al., 2009 [35]	Male Wistar rats	8	Infarct volume, neurological deficit score	Filament occlusion.	0.5 mA, 0.5 ms pulse width, 20 Hz	Right cervical vagus nerve.	VNS reduced infarct volume and improved functional score significantly.
Yang et al., 2022 [36]	Male Spontaneous hypertensive rats	16	IL-1B, Iba1 antibody level, a7nAchR level, neurodegeneration	Filament occlusion.	15 V, 1 ms pulse width, 25 Hz	Left cervical vagus nerve.	The stimulation reduced brain injury by downregulating the MMPs/IL-1β signalling pathway.
Li et al., 2020 [37]	Male Sprague–Dawley rats	8	Neurological deficit score, neuronal damage, infarct volume, micro vessel density, endothelial cell proliferative condition, and angiogenesis	MCAO/R	0.5 mA, 0.5 ms pulse width, 20 Hz	Left cavum concha	Auricular stimulation protected the brain from ischemic injury and was linked to angiogenesis activity. The effects of vagal stimulation were reduced by PPAR-γ silencing.
Jiang et al., 2016 [38]	Male Sprague-Dawley	8	Neurological deficit score, behavioral test, cerebral infarct volume, angiogenesis	MCAO/R	0.5 mA, 0.5 ms pulse width, 20 Hz	Left cavum concha	Auricular VNS provided significant neuroprotection and increased angiogenesis.
Ekici F et al., 2013 [39]	Male Wistar albino rats	7	neurological deficit score, infarct area	MCAO/R	1 mA, 500 µs pulse width, 20 Hz	Left cervical vagus nerve	VNS-treated animals had a higher neurologic score and a smaller infarct region. This was linked to higher antioxidant levels in brain samples.
Sun et al., 2012 [40]	Male Sprague-Dawley rats	8	Cerebral blood flow, infarct volume, Neurological score	PMCAO and MCAO/R	0.5 mA, 0.3 ms pulse width, 20 Hz	Right cervical vagus nerve	VNS neuroprotection against stroke in both temporary and permanent ischemia is unrelated to cerebral blood flow.
Jiang et al., 2015 [41]	Male Sprague-Dawley rats	6	Neurological deficit score, cerebral infarct volume, inflammatory markers	MCAO/R	0.5 mA, 0.5 ms pulse width, 20 Hz	Right cervical vagus nerve	PPARγ may participate in the process by which VNS modulates the neuro-inflammatory response following ischemia/reperfusion in rats.
Zhao et al., 2019 [42]	Male C57BL/6 mice	6	Infarct volume, neurological deficit score, neuronal apoptosis, microglial polarization	MCAO/R	25 Hz, 1 ms, 5 kHz sine waves average voltage of 15 V for 60 min	Left cer-vical va-gus nerve	Non-invasive VNS-mediated microglia activation via IL-17A signalling reduces neuronal apoptosis while promoting microglial M2 polarisation.
Li et al., 2020 [50]	Male Sprague–Dawley rats	8	Neurological recovery function, α7nAchR activation, axonal plasticity	MCAO/R	0.5 mA, 20 Hz, 0.5 ms pulse width	Left cavum conchae	Transcutaneous VNS increased the expression of a7nAchR in the ischemic cortex. This was linked to better neuro-behavioral performance and increased axonal plasticity.
Khodaparast et al., 2013 [43]	Female Sprague–Dawley rats	16	forelimb function, ischemic size	Endothelin −1 injection	0.8 mA, 100 μs pulse width, 30 Hz	Right cervical vagus nerve	In comparison to training alone, VNS combined with physical rehabilitation resulted in significant recovery of forelimb strength. The ischemic size was unaffected.
Hays et al., 2014 [44]	Female Sprague-Dawley rats	16	Forelimb strength	Endothelin −1 injection	0.8 mA, 100 µs pulse width, 30 Hz.	Left cervical vagus nerve	Vagus nerve stimulation administered after 2 h of forelimb training is less effective than VNS administered in conjunction with forelimb training.
Hays et al., 2016 [45]	Female Fisher rats	8–9	forelimb function, ischemic size	Endothelin −1 injection	0.8 mA, 100 μs pulse width, 30 Hz	Right cervical vagus nerve	In aged rats, VNS combined with rehabilitative training improves forelimb function recovery compared to rehabilitative training alone.
Khodaparast et al., 2014 [46]	Female Sprague-Dawley rats	10	Motor functions, infarct volume	Endothelin −1 injection	0.8 mA, 100 μs pulse width, 30 Hz	Right cervical vagus nerve	Combining VNS with motor rehabilitation can help with stroke recovery. During motor rehabilitation, VNS was used to restore rapid improvement and return to pre-lesion performance.
Khodaparast et al., 2016 [47]	Female Sprague-Dawley rats	9–10	Forelimb strength, lesion size	Endothelin −1 injection	0.8 mA, 100 μs pulse width, 30 Hz	Right cervical vagus nerve	VNS combined with rehabilitative training significantly improves forelimb function recovery. The benefits of VNS on forelimb function were maintained after stimulation was stopped. There are no differences in lesion size.
Meyers et al., 2018 [48]	Female Sprague-Dawley rats	6–8	Forelimb strength, lesion size	Endothelin −1 injection	0.8 mA, 100 ms pulse width, 30 Hz	Left cervical vagus nerve	VNS combined with rehabilitative training increases neuroplasticity in corticospinal motor networks to task-relevant musculature, which may provide insight into the neural changes that support VNS-dependent recovery improvement.

α7nAchR, α7-nicotinic acetylcholine receptor; Akt, Serine/Threonine-protein kinase; CD 68, Cluster of Differentiation 68; IL-1β, IBA-1, Ionized calcium-binding adapter molecule 1; Interleukin 1 beta; IL-6, interleukin 6; IL17A, Interleukine 17A; I/R, ischemic reperfusion; LPGDS, Lipocalin-type prostaglandin (PG) D synthase; MMPs, matrix metalloproteinase; MyD88, Myeloid differentiation primary response 88; PPARϒ, Peroxisome proliferator- activated receptor gamma; PMCAO, Permanent Middle Cerebral Artery Occlusion; TLR4, toll-like receptor 4; TNFα, tumour necrosis alpha; TMCAO, Transient Middle Cerebral Artery Occlusion, VNS, vagus nerve stimulation.

### 3.3. Prognostic Factors for Acute Stroke Ischemia

In an animal model, the neurological deficit score, infarct size, and forelimb strength strongly predict prognosis during post-cerebral ischemia.

A mean difference analysis of 18 studies with post-stroke behavioral examinations revealed a trend favoring cerebrovascular protection with VNS treatment compared with non-treated controls (Figure 2). The neurological deficit score was determined using a variety of scales, including 4-point, 5-point, 12-point, 18-point, and 20-point versions. They were all converted to four points for ease of comparison (Figure 2). The score was calculated 24 h after stroke induction. Compared with the control or ischemic reperfusion (I/R) groups, the neurological deficit score improved significantly in all studies.

Nineteen of the 29 studies measured infarct volume 24 h after stroke induction. Twelve studies reported a percentage reduction in infarct size before and after VNS (Figure 3), while the remaining seven reported results based on total volume reduction (Figure 4). An analysis of both types of reports showed that a reduction in infarct regions favors VNS intervention groups compared to the control.

**Figure 6:**
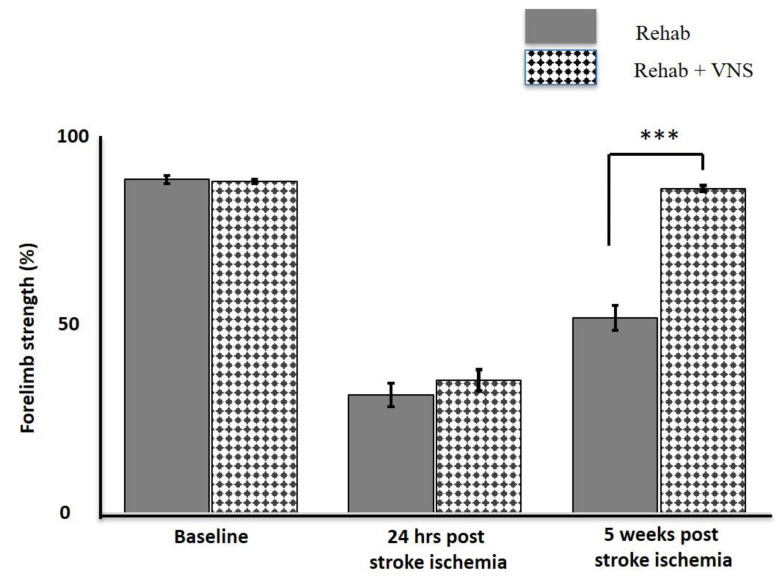
The average forelimb strength in the Rehab group (solid fill) and VNS along with Rehab group (pattern fill) over 5 weeks post stroke injury. The forelimb strength between groups were significantly different on the 5th weeks of VNS treatment and rehab *n* = 5. *** *p* < 0.001 [43,44,45,46,47].

Six studies examined the effects of VNS on forelimb strength, which is a major contributor to stroke disability (Figure 5). All six studies showed significant improvement in post-stroke forelimb strength when VNS was applied during rehabilitation (Rehab + VNS), surpassing rehabilitation therapy alone (Rehab Only). In five studies, forelimb strength after VNS treatment was nearly restored to baseline, while in one study by Meyers et al. [48], it did not return to normalcy but did show significant improvement. Due to the similarity in the research methodology between these studies, we extended our analysis by compiling data from each group (Rehab Only vs. Rehab + VNS) and examining the forelimb strength (Figure 6). While forelimb strength was nearly identical between the groups at baseline and post-stroke, the variation became apparent during post-treatment, with forelimb strength nearly returning to baseline.

### 3.4. Biomarker Analysis

Several biomarkers involved in stroke pathogenesis have been identified and are being studied for the neuroprotection elicited by VNS in stroke animals (Table 3). These include inflammatory cytokines, angiogenesis proteins, and anti-apoptotic proteins.

Eight of the 29 studies investigated inflammatory cytokines and reported the anti-inflammatory effects of VNS. Seven of the eight studies reported a decrease in TNF-α levels in the intervention group compared to the control group [21,23,26,28,29,30,41]. Yang, Bhaskar, Thompson, Duval, Torbey and Yang [36] found no evidence of a significant reduction in TNF-α levels. Six studies reported a decrease in IL-1β levels, and three studies found a reduction in IL-6 levels [21,29,41].

Three studies examined the expression of angiogenic factors, such as Vascular Endothelial Growth Factor (VEGF), Brain-derived Neurotrophic Factor (BDNF), Fibroblast Growth Factor 2 (FGF2), and endothelial NO· synthase (eNOS), in cerebral ischemic rat models following VNS. VEGF, one of the most important angiogenic factors, increased in all three studies. BDNF and eNOS were upregulated in two of the three studies, whereas FGF2 was upregulated only in the work by Long et al. [28,37,38].

Three studies have examined neuronal apoptosis in post-stroke ischemia in response to VNS. Zhang, Ma, Jin, Jia, Jiang and Li [49] found that VNS treatment significantly reduced the expression of the pro-apoptotic protein Bax, while increasing the expression of the anti-apoptotic proteins Bcl-2 and cleaved caspase-3 24 h after I/R injury. In two other studies, cleaved caspase 3 was downregulated and p-Akt was upregulated in response to VNS treatment [30,41].

**Table 3:** Summary of biomarker analysis.

Parameters	Biomarkers	Studies with Reported Effects of VNS		
		Increased	No Effect	Decreased
Inflammatory cytokines	TNFα	-	Yang, et al. [36]	* Zhao, et al. [29] * Jiang, et al. [30] * Xiang, et al. [21] * Jiang, et al. [41] * Ay, et al. [23] * Long, et al. [28] * Zhang, et al. [51]
	IL1β	-	Ay, et al. [30]	* Jiang, et al. [30] * Jiang, et al. [41] * Zhang, et al. [26] ** Long, et al. [28] ** Zhao, et al. [29] ** Yang, et al. [36]
	IL6	-	-	* Jiang, et al. [30] * Xiang, et al. [21] ** Zhang, et al. [33] ** Zhao, et al. [29]
Angiogenesis	VEGF	* Long, et al. [28] * Li, et al. [37] * Jiang, et al. [38]	-	-
	BDNF	* Li, et al. [37] * Jiang, et al. [38]	-	-
	FGF2	** Long, et al. [28]	-	-
	p-eNOS	* Jiang, et al. [38] * Li, et al. [37]	-	-
Apoptosis	Cleaved caspase 3	-	-	* Jiang, et al. [30], * Zhang, et al. [49]
	p-Akt	* Jiang, et al. [30] * Zhang, et al. [49]	-	-
	Bcl-2	* Zhang, et al. [49]	-	-

A summary of the biomarker analysis following VNS treatment. A double asterisk sign (**) denotes a significant difference between VNS and stroke induced animals (*p* < 0.01). A single asterisk sign (*) denotes a significant difference between VNS and stroke induced animals (*p* < 0.05). Bcl-2, B-cell lymphoma 2; BDNF, Brain Derived Neurotrophic Factor; FGF2, Fibroblast growth factor 2; IL-1β, Interleukin 1 beta; IL-6, Interleukin 6; p-Akt, phosphorylated Serine/Threonine-protein kinase; p-eNOS, Phospho-endothelial nitric-oxide synthase; TNFα, Tumour necrosis factor alpha; VEFG, Vascular endothelial growth factor.

## 4. Discussion

Overall, evidence from 29 studies suggests that VNS is an effective option for controlling stroke injury in rodents. Specifically, this review found a significant improvement in the neurological deficit score, infarct volume, forelimb strength, angiogenesis, and inflammation in studies in which VNS was applied. This is the first systematic review to assess the beneficial effects of VNS in animal models of stroke.

### 4.1. VNS Improves the Primary Indications of Stroke in Infarct Volume, Neurological Deficit Score, and Forelimb Strength

The assessment of the neurological deficit score is important in stroke rodent models because it aids in the evaluation of stroke outcomes. Although an early deficit helps predict the final outcome, the deficit changes frequently during the first few hours. Furthermore, there are different assessment time points, ranging from 24 h to 2 weeks. Therefore, we used 24 h as the time point of interest to achieve uniformity. It also allowed us to make pertinent comparisons between studies in terms of improving outcomes and predicting the best rehabilitation treatment. All 18 studies that measured neurological deficit scores showed improvement in response to VNS within 24 h (Figure 2).

Infarct volume represents the amount of brain damage following stroke induction. The measurement of infarct volume is a simple analysis that is frequently used in both animal research and clinical trials of stroke studies [52]. It is also useful for translational medicine research and helps in predicting treatment according to the extent of the damage [53]. For these reasons, infarct volume was primarily compared across VNS studies, with these studies measuring the infarct region 24 h after stroke induction. Our review included 19 studies that measured infarct volume and discovered that, after VNS intervention, infarct volume improved in all of them (Figure 3 and Figure 4). After VNS intervention, infarct size decreased by 25–50%, with Zhao et al., 2019 [42] demonstrating the greatest improvement.

This review also investigates the benefits of VNS on forelimb strength. Upper-limb motor impairment is one of the most common complications experienced by patients with ischemic stroke. Although few treatments are available to improve motor function, more than 60% of these patients who have been treated continue to have motor impairment [47]. In animal models of stroke, VNS combined with forelimb rehabilitation effectively restored motor function and plasticity (Figure 5). In these studies, the forelimb strength in rats was restored to baseline after co-VNS treatment and rehabilitation (Figure 6). Surprisingly, the rehabilitation-only groups showed less improvement than the VNS and rehabilitation combined groups. Similarly, VNS alone did not restore the pre-lesion forelimb strength [43,46,47]. However, because of the artificial induction of IS via Endothelin-1 (ET-1) injection into the perivascular surface of the middle cerebral artery, these findings should be interpreted with caution. It is well known that the presence of anesthesia can mask the stereotaxic injection of ET-1, resulting in a high degree of variability in stroke outcomes [54]. Interestingly, Hays et al. found that the improvement in forelimb function lasted even longer in aged rats even after stimulation was stopped, implying that VNS therapy might also be effective in the elderly population [45].

### 4.2. VNS Improves Stroke via the Nicotinic Anti-Inflammatory Pathway

Inflammation plays a significant role in the pathogenesis of ischemic stroke. The immune response begins locally in the occluded and hypo-perfused brain parenchyma, where inflammatory mediators are produced to clear necrotic tissues. Following reperfusion treatment, inflammatory responses are aggravated, resulting in ischemic reperfusion injury. The anti-inflammatory properties of VNS may help mitigate ischemia-reperfusion injury [36,41]. VNS potentially provides anti-inflammatory properties through its efferent fibers by activating the cholinergic anti-inflammatory pathway (CAP). When the vagus nerve is stimulated, acetylcholine (Ach) is released, inhibiting the anti-inflammatory pathway via the α7nAChR on activated macrophages and other cytokine-producing cells. The incubation of activated BV2 microglia with Ach triggers the phosphorylation of the Janus kinase 2/signal transducer and the activation of transcription 3 (JAK2/STAT3) and phosphoinositide 3 kinase/Akt (PI3k/Akt), inhibits the transformation of pro-inflammatory microglia (M1), and promotes the anti-inflammatory subtype (M2) [33]. In one study, M2 macrophages inhibited the release of pro-inflammatory cytokines, such as TNF, IL-6, and IL-1, while increasing the levels of anti-inflammatory factors, such as IL-4 and IL-10 [33]. In ischemic stroke models, VNS significantly reduced the release of pro-inflammatory cytokines in circulating plasma as well as their expression in the penumbra area [55,56].

### 4.3. Apoptosis

Apoptosis is usually determined by the balance of the pro-apoptotic proteins Bax and cleaved caspase 3 and the anti-apoptotic protein Bcl-2. Within a few hours of ischemic attack, anti-apoptotic Bcl-2 levels are reduced, whereas pro-apoptotic proteins are increased [57,58]. VNS therapy is effective in inhibiting neuronal apoptosis in stroke animals, as measured by the terminal deoxynucleotidyl transferase dUTP nick end labelling (TUNEL assay), and the reduction in pro-apoptotic proteins [59]. Furthermore, VNS therapy restored the Bcl-2:Bax protein ratio in the brain by increasing anti-apoptotic factors [57].

Besides inflammation, the PI3k/Akt signal transduction pathway has been extensively investigated for its role in regulating cellular apoptosis and survival [33]. Targeting the IP3/Akt signal transduction cascade may be a promising treatment for various ischemic reperfusion injuries [60], demonstrating that VNS increases Ach levels in the myocardial ischemic region and promotes Akt phosphorylation (p-Akt). VNS-treated animals showed higher p-Akt levels and less apoptotic damage in the heart [60]. Similar anti-apoptotic effects and p-Akt activation were also observed in an ischemic/reperfusion stroke model 24 h post-VNS treatment [34]. Importantly, p-Akt activation is associated with the inhibition of inflammatory and autophagic responses, implying that VNS treatment activates a plethora of interconnected neuroprotective responses [51].

### 4.4. Angiogenesis

Angiogenesis is a critical process that occurs after an ischemic stroke. Neovascularization post-IS restores cerebral blood flow within the ischemic penumbra to facilitate long-term functional recovery. Newly generated collateral blood arteries after focal cerebral ischemia can enhance the perfusion of the surrounding tissues and aid in restoring nervous system functions. Recent research suggests that angiogenesis aids in the restoration of brain function following ischemic stroke concurrently with neurogenesis [30,49,61]. There is evidence that the increase in cerebral blood volume seen in the late stages of stroke is caused by a surge in cerebral angiogenesis [49] and is crucial for the restoration of neuronal function in humans with stroke [61]. VNS not only improved neural symptoms and reduced infarct volume in cerebral I/R rats, but also increased the expression of angiogenic factors such as BDNF, eNOS, and VEGF, inducing endothelial cell proliferation, thus stimulating angiogenesis [38,49].

VEGF, one of the primary mediators of cerebral angiogenesis, begins to rise after stroke in humans. Neuronal cells secrete VEGF in response to stroke or hypoxic stress [62]. VEGF promotes angiogenesis and leads to functional recovery after a stroke. Some studies have found that angiogenesis is positively related to patient survival after stroke, implying that modulating vascular growth in injured tissue could be a critical therapeutic target [63,64]. One study found that, 21 days after stroke induction, the gene expression and protein levels of VEGF were higher in the VNS group than in the control group [38]. This finding suggests a potential role of VEGF in improving angiogenesis in response to VNS intervention.

eNOS and BDNF are two other angiogenic factors highlighted in our review. eNOS is a key integrator of the angiogenesis signaling pathway, which is frequently activated by Akt. The overexpression of eNOS increases vasodilation, promotes angiogenesis, mobilizes stem and progenitor cells, and improves vascular reactivity, all of which may improve endothelial cell function after ischemia/reperfusion [55]. The production of eNOS aids in the increase of circulating endothelial progenitor cells, which promotes post-stroke neovascularization [49]. BDNF is another important angiogenic factor that is expressed in both neural and non-neural tissues. Growth hormones and neurotrophic substances such as insulin or BDNF may activate Akt [65]. As a result, eNOS upregulation may be associated with increased BDNF levels via Akt activation [66]. BDNF also plays an important role in neural proliferation and cognitive function [67]. Decreased BDNF expression is reported to reduce endothelial cell survival [68,69]. In a study conducted on a rat model of cerebral ischemia, treatment with BDNF significantly reduced infarct size and neuronal cell death [70]. In another Middle Cerebral Artery Occlusion (MCAO) model of stroke, auricular VNS enhanced BDNF gene expression in the rat brain, suggesting that it is one of the factors that promotes angiogenesis [38]. Overall, we could say that VNS helps in neo-angiogenesis or enhances angiogenesis factors, which leads to reduced infarct size, reduced neuronal damage, and improved neuronal function, thus providing neuroprotection in VNS ischemic stroke models.

### 4.5. Limitations and Future Directions

VNS can be utilized to treat patients with stroke owing to its efficacy in stroke-induced animal models, as shown in our review. However, this therapeutic option must overcome significant obstacles before transitioning from preclinical research to clinical application in human participants. Studies using experimental stroke models have helped us understand some of the mechanisms underlying VNS treatment; however, clinical trials using VNS as an independently administered therapy for patients with stroke have not yielded highly successful results [71,72]. This is a well-known problem in biomedical research (that is, the applicability of any medical procedure to humans). A pertinent example is applicable with VNS; while studies in animal models have successfully reduced stroke symptoms, clinical studies in individuals with ischemic stroke [49] and endotoxemia do not support the anti-inflammatory and neuroprotective effects of VNS [61]. This discrepancy may be attributed to the anatomical variations between rats and humans. A quantitative morphological analysis revealed that the cervical vagus nerve in rats was 10 times smaller in diameter than in humans, had fewer fascicles, and had a thinner perineurium, resulting in a variation in the activation threshold upon direct electrical stimulation [73]. The injection of the retrograde tracer cholera toxin B into the common transcutaneous VNS site (that is, the tragus) in rats resulted in dense labelling in the dorsal horn of the upper cervical spinal cord, but limited labelling in the nucleus tractus solitarius (NTS) [74]. In contrast, functional neuroimaging studies in humans have shown that activation is concentrated in the NTS region, also known as the vagal afferent integration center [75]. The use of non-uniform stimulation parameters in either animal or human studies causes the translation of VNS to become more complex. Recent research found that stimulating the auricular VNS at 15 Hz resulted in greater anti-inflammatory responses than at 25 Hz, indicating the importance of having standardized stimulation parameters in a VNS study [76]. While we demonstrated the protective effects of VNS in an animal stroke model, we admit that our comparison of different VNS stimulation designs and parameters was flawed (Table 2). For a better analysis, future studies should consider removing the covariables and differentiate between invasive and non-invasive VNS.

Another limitation of this study was that it compared parameters only at 24 h post-stroke induction. Twenty-four hours were taken in this review as the time for comparison among studies, as most of the studies measured parameters at that time, thus making our comparison easier and consistent. In future animal studies, researchers could observe a time where there is most improvement in outcomes; a study has demonstrated that neurological deficit scores improve significantly 7 days and 14 days after VNS compared to 24 h, thus suggesting that these time points are more relevant and effective [21]. In addition, in this review, most model animals were male, which is another problem in preclinical studies and hinders its translation to human studies [77]. Moreover, with respect to stroke, it occurs more frequently in females; thus, female models would provide more aid in developing and translating the treatment [78]. Therefore, future studies should be conducted in females to limit sex bias.

In clinical practice, reperfusion therapy remains the gold standard therapy for stroke patients [3,4]. One question to consider is whether VNS therapy is more efficient than just reperfusion in an animal model. However, extrapolating from the existing literature is difficult because previous studies were designed to investigate the potential use of VNS in treating ischemic reperfusion injury in stroke animals. For example, the reperfusion alone group is considered to be the control group, whereas the reperfusion and VNS group is considered the treatment group. Nonetheless, Sun et al. (2012) [40] found that both VNS alone and VNS combined with reperfusion improved infarct volume but not neurological function in stroke animals, suggesting the differential effects between these treatments.

## 5. Conclusions

**Figure 7:**
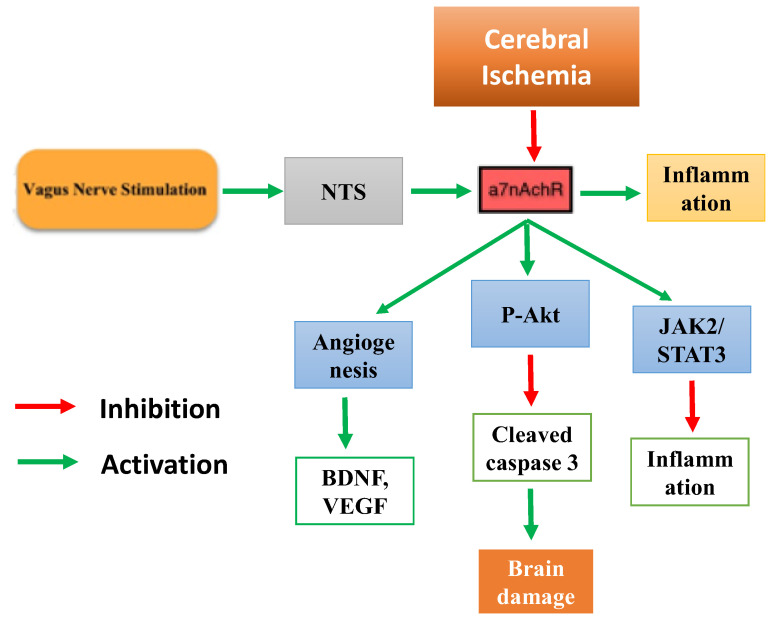
Vagus nerve stimulation has shown to improve stroke with various proposed mechanisms such as anti-apoptosis, angiogenesis and anti-inflammation. NTS—Nucleus of the solitary tract, a7nAChR—a7-nicotinic acetylcholine receptor, BDNF—Brain Derived Neurotrophic Factor, VEGF—Vascular endothelial growth factor, p-Akt—phosphorylated protein kinase B.

Our current review demonstrates that VNS provides neuroprotection and is effective in improving post-ischemic stroke symptoms in animal models of stroke. VNS treatment improves the neurological deficit score and infarct volume, which reflects the severity of stroke. Furthermore, VNS reduces the levels of inflammatory, apoptotic, and increased angiogenic markers, which aids in the prediction of post-stroke recovery (Figure 7). Interestingly, co-treatment with VNS and physical rehabilitation results in significant recovery compared with rehabilitation alone. The assimilation of comparative animal models in this review may help researchers design future VNS studies in humans.

## Figures and Tables

**Table 1:** Rating the risk of bias for studies included in the analysis [20].

Study	Selection Bias	Performance Bias	Attrition/Exclusion Bias	Detection Bias	Selective Reporting Bias
Xiang et al. 2015 [21]	++	−	+	+	++
Ay et al. 2015 [22]	++	++	+	+	++
Ay et al. 2016 [23]	++	++	+	−	++
Yang et al. 2018 [24]	++	++	+	++	+
Lindemann et al. 2020 [25]	++	+	+	+	+
Zhang et al. 2021 [26]	++	++	+	++	++
Lu et al. 2017 [27]	++	+	+	+	++
Long et al. 2022 [28]	++	++	+	+	++
Zhao et al. 2022 [29]	++	++	−	+	+
Jiang et al. 2014 [30]	+	++	+	+	++
Hiraki et al. 2012 [31]	++	+	+	+	++
Ay et al. 2011 [32]	+	+	+	+	+
Zhang et al. 2017 [33]	+	++	+	++	++
Jiang et al. 2015 [34]	++	++	+	++	++
Ay et al. 2009 [35]	++	++	+	+	++
Yang et al. 2022 [36]	++	+	+	+	+
Li et al. 2020 [37]	+	+	+	−	++
Jiang et al. 2016 [38]	+	+	+	−	++
Ekici et al. 2013 [39]	++	++	+	+	++
Sun et al. 2012 [40]	++	+	+	−	++
Jiang et al. 2015 [41]	++	++	+	−	++
Zhao et al. 2019 [42]	++	++	+	+	++
Li et al. 2020 [37]	++	++	+	++	++
Khodaprast et al. 2013 [43]	++	++	+	+	++
Hays et al. 2014 [44]	++	++	+	++	++
Hays et al. 2016 [45]	++	++	+	++	++
Khodaparast et al. 2014 [46]	++	++	+	++	++
Khodaparast et al. 2016 [47]	+	−	+	−	++
Meyers et al. 2018 [48]	++	+	+	++	+

Summary of risk of bias judgments for the in vitro and in vivo studies that were included in our analysis using the Office of Health Assessment and Translation (OHAT) framework. Color coding is defined as green (definitely low bias), yellow (probably low bias), and red (probably high bias). A double plus sign (++) indicates a very high study quality, a single plus sign (+) indicates a high study quality, and a single minus sign (−) indicates a low study quality.

## Data Availability

Data sharing is not applicable to this article.
